# Safety and efficacy of bone marrow mononuclear cell therapy for ischemic stroke recovery: a systematic review and meta-analysis of randomized controlled trials

**DOI:** 10.1007/s10072-023-07274-x

**Published:** 2024-01-03

**Authors:** Yanbing Tang, Zilan Wang, Haiying Teng, Hanyu Ni, Huiru Chen, Jiaye Lu, Zhouqing Chen, Zhong Wang

**Affiliations:** 1 Department of Neurosurgery & Brain and Nerve Research Laboratory, The First Affiliated Hospital of Soochow University, 188 Shizi Street, Suzhou, 215006 Jiangsu Province China; 2 Suzhou Medical College of Soochow University, Suzhou, 215002 Jiangsu Province China; 3 Department of Neurology, The First Affiliated Hospital of Soochow University, 188 Shizi Street, Suzhou, 215006 Jiangsu Province China

**Keywords:** Stroke, Cell-based therapy, Bone marrow mononuclear cells, Meta-analysis, Systematic review

## Abstract

**Background:**

Cell-based therapy represents a potential treatment for ischemic stroke (IS). Here, we performed a systematic review and meta-analysis to summarize the evidence provided by randomized controlled trials (RCTs) for the transplantation of bone marrow mononuclear cells (BMMNCs) in patients with IS in any phase after stroke.

**Methods:**

We searched several databases for relevant articles up to the 10th of March 2023, including MEDLINE, EMBASE, the Cochrane Library, and ClinicalTrials.gov. Subgroup analyses were implemented to evaluate the dose and route of BMMNC administration. Statistical data were analyzed by Review Manager version 5.3 software.

**Results:**

Six RCTs were included in this article, including 177 patients who were treated by the transplantation of BMMNCs and 166 patients who received medical treatment. The three-month National Institutes of Health Stroke Scale (NIHSS) score indicated a favorable outcome for the BMMNC transplantation group (standardized mean difference (SMD), − 0.34; 95% confidence interval (CI), − 0.57 to − 0.11; *P* = 0.004). There were no significant differences between the two groups at six months post-transplantation with regards to NIHSS score (SMD 0.00; 95% CI − 0.26 to 0.27; *P* = 0.97), modified Rankin Scale (risk ratio (RR) 1.10; 95% CI 0.75 to 1.63; *P* = 0.62), Barthel Index change (SMD 0.68; 95% CI − 0.59 to 1.95; *P* = 0.29), and infarct volume change (SMD − 0.08; 95% CI − 0.42 to 0.26; *P* = 0.64). In addition, there was no significant difference between the two groups in terms of safety outcome (RR 1.24; 95% CI 0.80 to 1.91; *P* = 0.33).

**Conclusion:**

Our meta-analysis demonstrated that the transplantation of BMMNCs was safe; however, the efficacy of this procedure requires further validation in larger RTCs.

**Supplementary Information:**

The online version contains supplementary material available at 10.1007/s10072-023-07274-x.

## Introduction

Stroke is the second leading cause of death worldwide and a major cause of disability [1]. The lifetime risk of stroke in individuals over the age of 25 years is estimated to be 24.9% globally; of those experiencing stroke, the risk of ischemic stroke (IS) is 18.3% [2]. When suffering a stroke, cerebral blood vessels become blocked due to the formation of blood clots. This can lead to cerebral edema and cerebral infarction, causing irreversible damage to neurological function and seriously affecting a patient’s prognosis [3].

Early treatments, such as intravenous thrombolysis and arterial embolization, have been shown to significantly reduce the mortality and disability rate after suffering acute IS although the time window for treatment is restricted to only 6 to 8 h [4–6]. In the subacute to chronic phases of IS, treatment strategies may be adjusted depending upon an individual patient’s condition. These strategies may include the management of cholesterol, antithrombotic medications, and rehabilitation training; however, these methods are associated with limited efficacy in the promotion of functional recovery [7]. IS largely considered to have a poor prognosis, and no therapies have been proven to treat this condition effectively. However, the advancement of cell transplantation technology has provided a new avenue for the management of IS in the acute, subacute, or chronic phase [8].

Cell-based therapies for stroke emerged in the 1990s and are widely considered to represent a potential treatment for IS [9, 10]. Cell-based therapy can improve the prognosis of patients with IS by facilitating cell replacement, stimulating endogenous repair processes, promoting brain plasticity and synaptic reorganization, and facilitating immunomodulation [11, 12]. Currently, cell-based therapy research includes the use of different types of cells, such as bone marrow mononuclear cells (BMMNCs), hematopoietic stem cells, neural stem cells, and mesenchymal stem cells; different routes of administration, such as intracerebral, intra-arterial, intravenous, intrathecal, and intranasal; different doses, depending on cell type and the route of administration; and different time windows for treatment, ranging from days to months and years [13]. Of the various cell types, hematopoietic stem cells have been shown to possess only limited ability to differentiate into neurons; neural stem cells have been associated with ethical issues and immune-related problems, and mesenchymal stem cells require several weeks for expansion with conventional culture techniques [14]. BMMNCs offer significant potential since they can be obtained from the patients themselves without expansion and are easy to collect, prepare, and preserve, thus avoiding potential ethical issues or immune-related problems.

BMMNCs represent a heterogeneous mix of hematopoietic progenitor cells, a population of mesenchymal and endothelial precursors [15]. Previous research has demonstrated that these cells have the capacity to protect neurons and reduce the loss of neurons resulting from stroke [16, 17]. The autologous transplantation of BMMNCs has been shown to be efficacious in animal models of stroke by exerting a range of biological effects, including the attenuation of neuronal death, the modulation of microglia, the reduction of proinflammatory responses, the enhancement of neoangiogenesis, and the promotion of endogenous neural stem cell proliferation [18, 19]. The mechanisms aimed at enhancing the outcomes of stroke may differ according to the specific phase of stroke. During the acute phase, BMMNC therapy facilitates neuroprotection mainly by releasing trophic factors, regulating inflammation, and promoting neurorestoration. In the chronic phase, the focus transitions to neurorestoration in patients with a stable chronic deficit [20].

However, the efficacy of BMMNC transplantation remains controversial, and numerous key parameters have yet to be fully determined, including the time window for treatment, dosage, and the route of BMMNC administration. Furthermore, there are no published meta-analyses of randomized controlled trials (RCTs) relating to the transplantation of BMMNCs in patients with IS. Therefore, in the present study, we conducted a meta-analysis to summarize existing evidence for the efficacy and safety of BMMNC transplantation in IS.

## Methods

### Study protocol

Prior to commencing the project, we drafted a research protocol following the Cochrane Collaboration format [21], which was registered on the INPLASY website (Register number: INPLASY202340061 https://inplasy.com/inplasy-2023-4-0061/).

### Eligibility criteria

Our inclusion criteria were as follows: (1) participants (adults diagnosed with IS); (2) intervention (the transplantation of BMMNCs with medical treatment at any phase of IS); (3) comparison group (conventional medical treatment); and (4) outcome (the main outcome was the National Institutes of Health Stroke Scale (NIHSS) score at three months while the secondary outcomes included NIHSS scores at six months, modified Rankin Scale (mRS) scores at six months, Barthel Index (BI) scores at six months, and changes in infarct volume at six months). Safety outcomes included the occurrence of adverse events, including partial seizures, fever, infection, and vascular disorders; and (5) study type: RCT.

Publications were excluded based on the following criteria: (1) case reports, case series, comments, letters, reviews, retrospective studies, prospective studies, or animal experiments, and (2) publications featuring incomplete information or lacking extractable data.

### Search strategy

Two dependent investigators (YBT and ZLW) searched MEDLINE, EMBASE, the Cochrane Library, and ClinicalTrials.gov to identify related articles up to the 10th of March 2023. We used a range of keywords (including “stem cell,” “bone marrow mononuclear cell,” and “stroke”) to develop different search strategies for different databases. The detailed search strategies are shown in Table S1.

### Quality assessment and data collection

The risk of bias for the included RCTs was assessed by applying the Cochrane Collaboration tool [22]. The quality of the attained outcome was assessed using the GRADEpro Guideline Development Tool. The quality of the articles was independently assessed by two researchers (YBT and ZLW), with differences resolved by a third researcher (HYT). After evaluating and identifying the articles, two authors (HYN and HRC) extracted a range of data from each of the included RCTs, including the name of the first author, year of publication, publication, study region, intervention, the number of included patients, patient age, sex ratio, and outcome events, as summarized in Table 1.

**Table 1:** Characteristics of the included studies and outcome events

Study	Publications	Countries	Study design	Centers	Regimen	Phase	Treatment group (no. of participants)	Male (%)	Mean age (years) ± SD	Outcome Events
Moniche 2023	Lancet Neurol	Spain	Open-label, RCT (NCT02178657)	4	Intra-arterial injection of 2 × 10^6^ BMMNCs/kg or 5 × 10^6^ BMMNCs/kg	Acute	BMMNC: 39 Control: 38	BMMNC: 54 Control: 66	BMMNC: 64.46 ± 15.22 Control: 66.0 ± 13.3	a, b, c, e, f
Savitz 2019	Circulation	USA	RCT (NCT01273337)	10	Intra-arterial infusion of 5 × 10^7^ BMMNCs (two cycles)	Subacute	BMMNC: 29 Control: 19	BMMNC: 69 Control: 94	BMMNC: 59.3 ± 10.03 Control: 62. 9 ± 10.81	a, b, d, f
Bhatia 2018	AJNR Am J Neuroradiol	India	Open-label, blinded-end point, RCT	1	Intravenous injection of a maximum of 5 × 10^8^ BMMNCs	Subacute	BMMNC: 10 Control: 10	BMMNC: 80 Control: 60	BMMNC: 57 ± 12.2 Control: 66 ± 7.3	c, f
Jin 2017	Int J Clin Exp Med	China	RCT	1	Subarachnoid injection of 1 × 10^7^ BMMNCs	Chronic	BMMNC: 10 Control: 10	BMMNC: 90 Control: 60	BMMNC: 50.80 ± 17.428 Control: 53.10 ± 13.068	a, b, c, f
Liu 2014	Chinese Journal of Physical Medicine and Rehabilitation	China	RCT	1	Subarachnoid injection of 1 × 10^7^ BMMNCs/kg (four cycles)	Subacute	BMMNC: 29 Control: 29	BMMNC: 62 Control: 69	BMMNC: 55.34 ± 3.63 Control: 56.87 ± 4.39	a, f
Prasad 2014	Stroke	India	Single blinded, RCT (NCT01501773)	5	Intravenous injection of 2.8 × 10^8^ BMMNCs	Subacute	BMMNC: 60 Control: 60	BMMNC: 68 Control: 60	BMMNC: 50.7 ± 11.6 Control: 52.5 ± 12.1	a, b, c, d, e, f

*RCT* randomized controlled trial, *SD* standard deviation (year), *BMMNC* bone marrow mononuclear cell, *a* 3-mounth NIHSS, *b* 6-month NIHSS, *c* 6-month mRS, *d* 6-mounth BI change, *e* infarct volume change, *f* adverse events

### Outcome measures

We included the NIHSS score at three months as the primary outcome, NIHSS, mRS, BI score, and infarct volume change at six months as secondary outcomes since they represent the major scales for measuring neurological impairment and the prognosis of stroke. The mRS is used to measure the neurological recovery of stroke patients on a scale of seven points from 0 to 6 [23]. The NIHSS score ranges from 0 to 42 and is used to assess the degree of functional impairment caused by stroke; this consists of a total of 11 tests, with higher scores indicating more severe neurological impairment [24]. The BI scale is used to assess a patient’s ability to perform activities of daily living and can be used to evaluate functional recovery before and after treatment. The BI consists of ten items with a total score of 100; independent ability is positively correlated with the BI score [25]. For the safety endpoint, we analyzed the occurrence of adverse events within each RCT.

### Subgroup analysis

We performed subgroup analysis based on the dose and route of administration. After examining the doses of all trials, we defined an injection of ≥ 3 × 10^6^ BMMNCs/kg as a high dose and < 3 × 10^6^ BMMNCs/kg as a low dose. We also categorized the injection methods; three RCTs involved intra-arterial injections [20, 26, 27], one RCT involved intravenous injections [28], and two RCTs involved subarachnoid injections [29, 30].

### Statistical analyses

Review Manager version 5.3 software was used to conduct all statistical analyses. For continuous variables, we calculated the standardized mean difference (SMD) and 95% confidence interval (CI). For dichotomous results, we used the risk ratio (RR) with 95% CIs. Cochrane’s Q test and the *I*^*2*^ statistic were calculated to access heterogeneity. A random-effects model was used for data with significant heterogeneity (*P* ≤ 0.1 and *I*^*2*^ ≥ 50%) while a fixed-effects model was used for data without significant heterogeneity (*P* > 0.1 and *I*^*2*^ < 50%). A *P* value < 0.05 was considered to be statistically significant.

## Results

### Search results and study characteristics

**Fig. 1:**
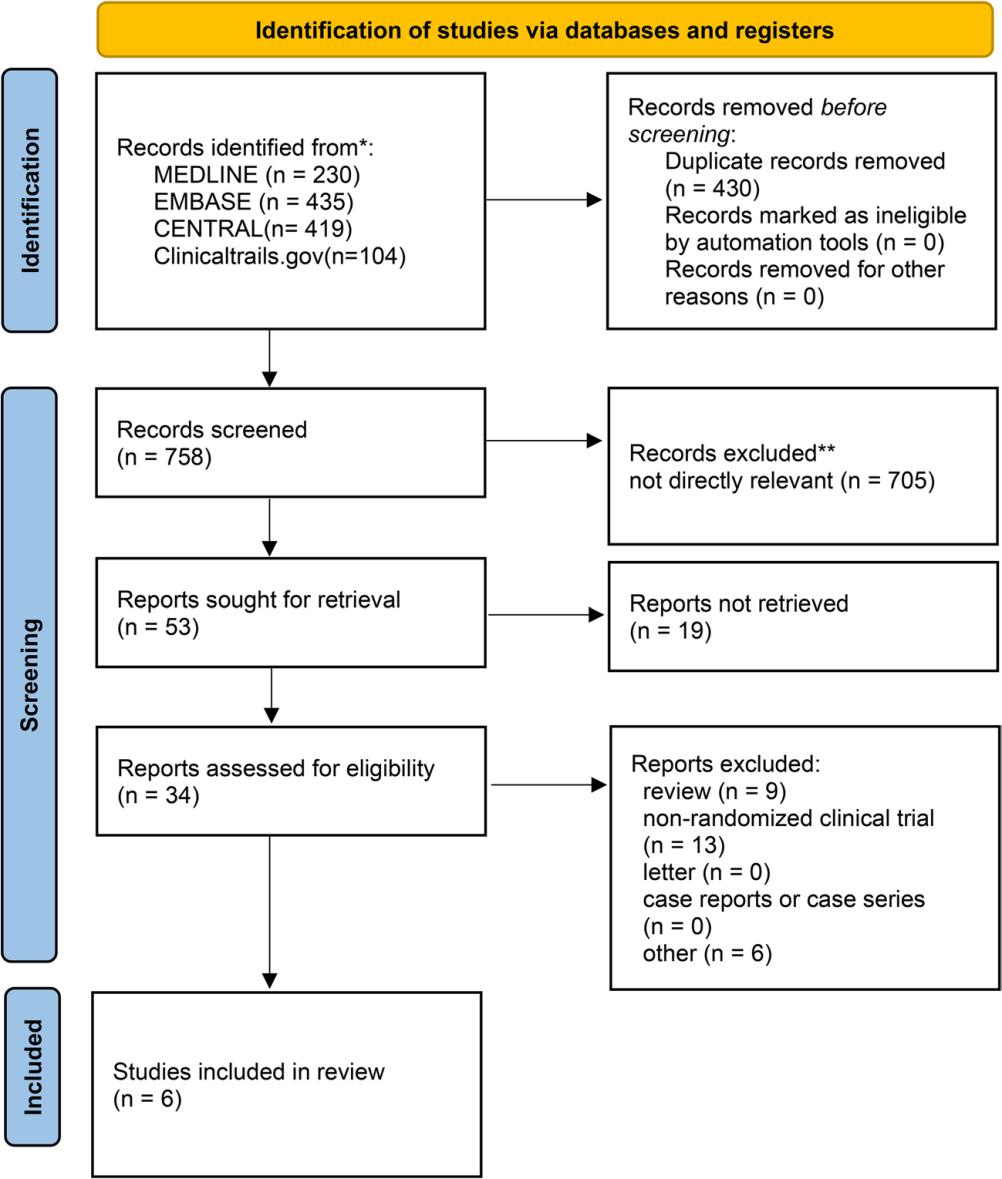
The study search, selection, and inclusion process

We initially identified 1188 articles from Medline, Embase, the Cochrane Library, and ClinicalTrials.gov. Of these, 430 duplicate articles and 705 articles that were not directly relevant were excluded. After scanning 53 articles, 19 articles were excluded for not retrieved, and 28 articles were excluded because they were in inappropriate formats, including reviews, non-randomized clinical trials, case reports, and letters. Finally, six RCTs [20, 26–31] were included in our final meta-analysis. Figure 1 shows a flowchart illustrating the process used for literature searches.

The main characteristics of the six included studies are listed in Table 1. Four of the research studies were from Asia (two from China, and two from India) [26, 28–31], one study was from Spain [20], and one study was from the USA [27]. The six studies included a total of 343 patients;177 patients were transplanted with BMMNCs; and 166 patients received medical treatment.

### Efficacy outcome analysis

**Fig. 2:**
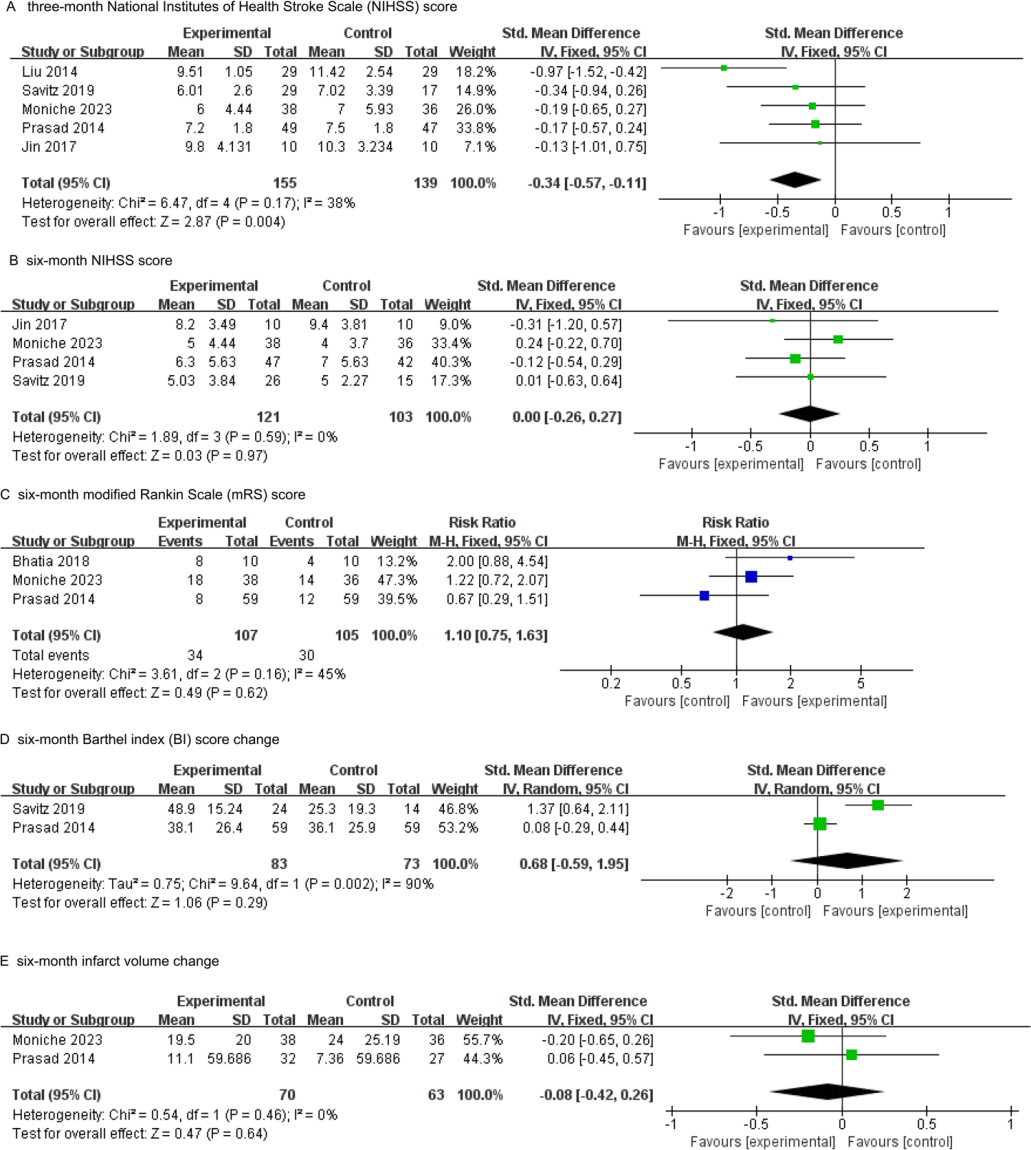
Meta-analysis of efficacy: **A** Three-month National Institutes of Health Stroke Scale (NIHSS) score. **B** Six-month NIHSS score. **C** Six-month modified Rankin scale (mRS) score. **D** Six-month Barthel index (BI) score change. **E** Six-month infarct volume change

The primary outcome was the NIHSS score at three months. Five studies reported NIHSS scores at three months; there was a significant difference between the BMMNC transplantation group and the control group (SMD − 0.34; 95% CI − 0.57 to − 0.11; *P* = 0.004; *I*^*2*^ = 38%; Fig. 2A).

The secondary outcomes were the NIHSS score, the mRS score, the change in BI score, and the change in infarct volume at six months. Four studies reported the NIHSS score at six months after the transplantation of BMMNCs; there was no significant difference between the treatment group and the control group (SMD 0.00; 95% CI − 0.26 to 0.27; *P* = 0.97; *I*^*2*^ = 0%; Fig. 2B). Three studies investigated the number of patients with favorable clinical outcomes (mRS scores ≤ 2) within six months of BMMNC transplantation; there was no significant difference compared to the control group (RR 1.10; 95% CI 0.75 to 1.63; *P* = 0.62; *I*^*2*^ = 45%; Fig. 2C). There was no significant difference between the control group and the BMMNC transplantation group in terms of the change of BI at six months (SMD 0.68; 95% CI − 0.59 to 1.95; *P* = 0.29; *I*^*2*^ = 90%; Fig. 2D). There was no significant difference between the BMMNC transplantation group and the control group in terms of the change of infarct volume at six months (SMD − 0.08; 95% CI − 0.42 to 0.26; *P* = 0.64; *I*^*2*^ = 0%; Fig. 2E).

### Safety outcome analysis

**Fig. 3:**
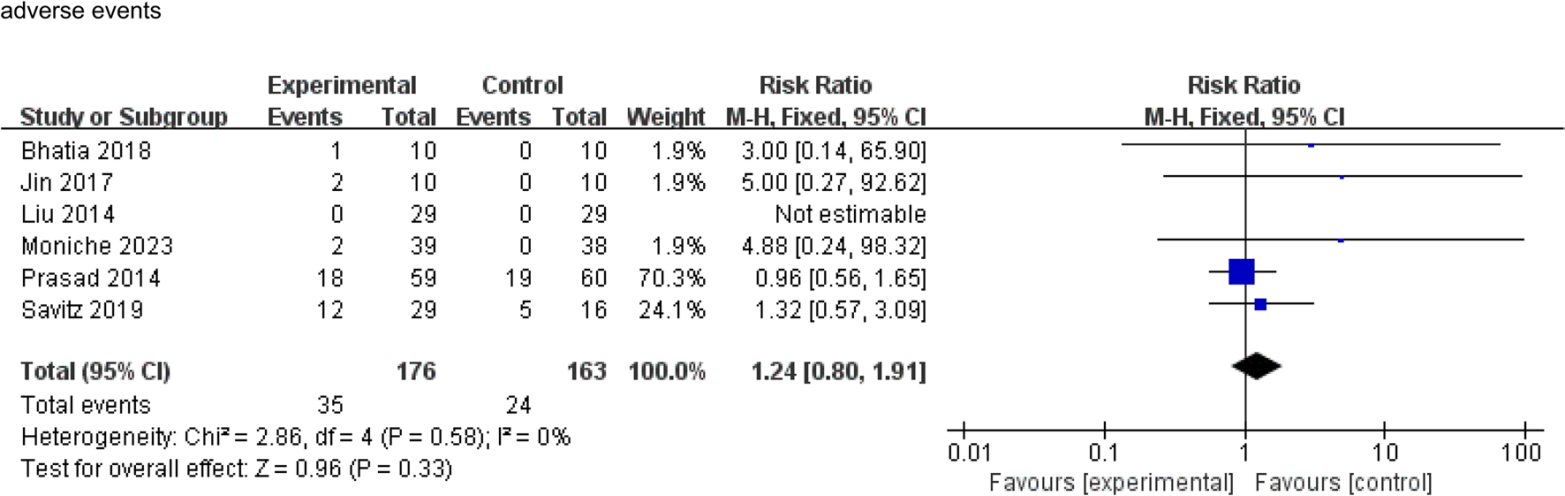
Meta-analysis of safety: adverse events

With regard to safety outcome, there was no significant difference between the BMMNC transplantation group and the control group in terms of the number of patients experiencing adverse effects, such as fever, infection, seizures, and cardiac disorders (RR 1.24; 95% CI 0.80 to 1.91; *P* = 0.33; *I*^*2*^ = 0%; Fig. 3).

### Subgroup analysis

We also conducted subgroup analysis to investigate the effect of different cell doses and routes of administration. We found no significant difference for different doses in terms of NIHSS score, mRS score, and the change of infarct volume (Table 2). However, in terms of the change in BI score at six months, we observed a significantly better result for low doses of BMMNCs (SMD 1.37; 95% CI 0.64 to 2.11; *P* = 0.0003; Table 2); however, this result should be interpreted with caution since only one RCT was included in this subgroup. With regard to the route of administration, we observed a more significant effect for the subarachnoid route in terms of the improvement of NIHSS scores at three months after treatment (SMD − 0.73; 95% CI − 1.20 to − 0.27; *P* = 0.002; *I*^*2*^ = 61%; Table 2). We also identified a more significant effect for the intra-arterial route with respect to the change of BI score at six months (SMD 1.37; 95% CI 0.64 to 2.11; *P* = 0.0003; Table 2); however, this analysis also included one RCT.

**Table 2:** Subgroup analysis of outcomes

	Efficacy outcomes												Safety outcomes	
	mRS at six months				NIHSS at three months		NIHSS at six months		BI change at six months		Infarct volume change at six months		Adverse events	
	RR (95%CI)	*P* value	SMD (95% CI)	*P* value	SMD (95% CI)	*P* value	SMD (95% CI)	*P* value	SMD (95% CI)	*P* value	SMD (95% CI)	*P* value	RR (95%CI)	*P* value
**1. Dose of BMMNCs**														
High	1.15 (0.64, 2.08)	0.64	N/A	N/A	− 0.37 (− 0.92, 0.18)	0.19	0.19 (− 0.15, 0.53)	0.28	0.08 (− 0.29, 0.44)	0.68^*^	− 0.12 (− 0.50, 0.26)	0.54	1.02 (0.60, 1.72)	0.95
Low	N/A	N/A	− 0.45 (− 1.59, 0.68)	0.43	− 0.31 (− 0.68, 0.06)	0.10	− 0.05 (− 0.43, 0.32)	0.78	1.37 (0.64, 2.11)	0.0003*	− 0.24 (− 0.79, 0.31)	0.39^*^	1.59 (0.71, 3.56)	0.26
**2. Route of administration**														
Intra-arterial	1.39 (0.89, 2.16)	0.15	N/A	N/A	− 0.24 (− 0.61, 0.12)	0.19	0.16 (− 0.21, 0.53)	0.39	1.37 (0.64, 2.11)	0.0003^*^	− 0.20 (− 0.65, 0.26)	0.40^*^	1.68 (0.76, 3.69)	0.20
Intravenous	0.67 (0.29, 1.51)	0.33^*^	N/A	N/A	− 0.17 (− 0.57, 0.24)	0.42^*^	− 0.12 (− 0.54, 0.29)	0.56^*^	0.08 (− 0.29, 0.44)	0.68*	0.06 − 0.45, 0.57)	0.81^*^	0.96 (0.56, 1.65)	0.89*
Subarachnoid	N/A	N/A	0.10 (− 0.78, 0.98)	0.83^*^	− 0.73 (− 1.20, − 0.27)	**0.002**	− 0.31 (− 1.20, 0.57)	0.49^*^	N/A	N/A	N/A	N/A	5.00 (0.27, 92.62)	0.28

*mRS* modified Rankin Scale, *NIHSS* National Institutes of Health Stroke Scale, *BI* Barthel index, *SMD* standardized mean difference, *RR* risk ratio, *CI* confidence interval, *BMMNC* bone marrow mononuclear cells, *N/A* not applicable

^*^Only one study was included

Bolded *P* values indicate statistically significant findings (* P* < 0.05)

### Risk of bias

**Fig. 4:**
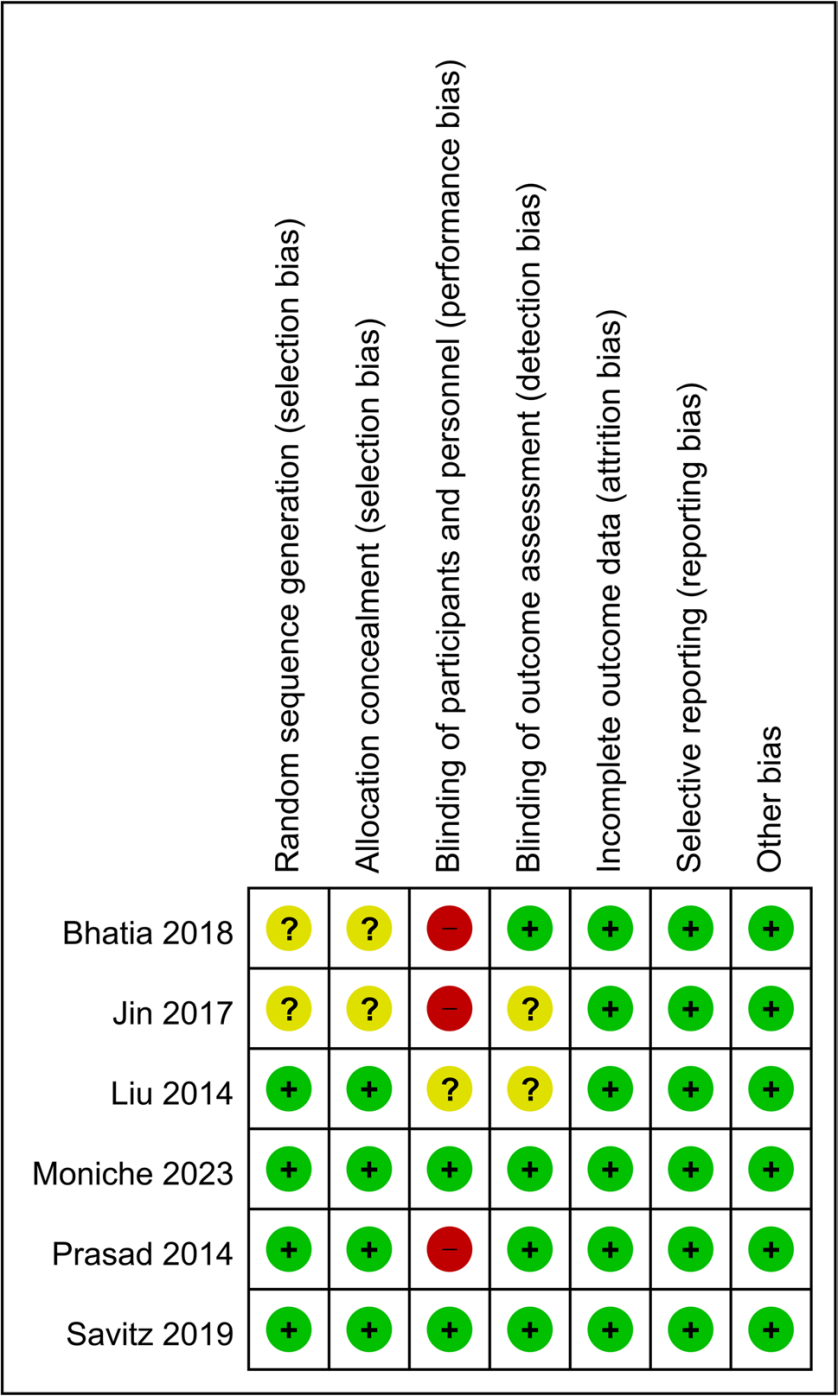
Risk of bias: a summary table for each risk of bias item for each study

Details relating to the risk of bias assessment for the included RCTs are shown in Fig. 4. Despite the assessment results of some articles being unclear, three articles had higher risks in terms of performance bias since they did not use sham injections; this may have resulted in the failure to blind all patients due to the obvious differences between BMMNC transplantation injection and conventional medical therapy. The quality of GRADE evidence is summarized in Table 3. With regard to the NIHSS, mRS, BI, and infarct volume, the quality of evidence varied from low to moderate.

**Table 3:** GRADE evidence profile of BMMNCs transplantation in patients with ischemic stroke

Outcomes	Participants (studies)	Overall certainty of evidence	Relative effect (95% CI)	Anticipated absolute effects	
				Risk with control group	Risk difference with BMMNCs transplantation
3-month NIHSS	294 (5 RCTs)	⨁⨁◯◯ Low^a,b^	-	The mean NIHSS was 8.65	SMD 0.34 SD lower (0.57 lower to 0.11 lower)
6-month NIHSS	224 (4 RCTs)	⨁⨁◯◯ Low^a,b^	-	The mean NIHSS was 6.35	SMD 0 SD (0.26 lower to 0.27 higher)
6-month mRS	192 (3 RCTs)	⨁⨁⨁◯ Moderate^a^	RR 1.15 (0.64 to 2.08)	286 per 1,000	43 more per 1,000 (103 fewer to 309 more)
6-month BI change	156 (2 RCTs)	⨁⨁⨁◯ Moderate^b^	-	The mean BI was 31.0	SMD 0.68 SD higher (0.59 lower to 1.95 higher)
6-month infarct volume change	133 (2 RCTs)	⨁⨁⨁◯ Moderate^b^	-	The mean infarct volume change was 15.68	SMD 0.08 SD lower (0.42 lower to 0.26 higher)

*CI* confidence interval, *SMD* standardized mean difference, *RCT* randomized clinical trial, *RR* risk ratio, *BMMNC* bone marrow mononuclear cell, *NIHSS* National Institutes of Health Stroke Scale, *mRS* modified Rankin Scale, *BI* Barthel Index

^a^Risk of selection and performance bias within studies

^b^Presence of heterogeneity between studies

## Discussion

We conducted a meta-analysis related to the treatment of IS with BMMNC transplantation based on six RCTs. Our study demonstrated that the transplantation of BMMNCs resulted in a statistically significant improvement in NIHSS scores at three months when compared with control groups. In addition, the transplantation of BMMNCs did not increase the incidence of adverse effects. However, no statistically significant differences were observed in terms of NIHSS scores, mRS scores, BI scores, or changes in infarct volume, when compared between the BMMNC transplantation group and the control group at six months.

The latest Global Burden of Disease (GBD) 2019 stroke burden reported that the mortality rate of stroke was estimated to be 4.53% [32]. Over the last ten years, the application of cell-based therapy for stroke has advanced from bench to bedside. Among the various categories of cell-based therapies, BMMNCs derived from the patient’s own bone marrow have emerged as a particularly promising method, as evidenced by several studies incorporating animal models [15, 33–35]. BMMNCs enhance endogenous recovery mechanisms both locally and in distant locations from the infarct, potentially via immunomodulation and the reduction of post-stroke inflammation. Both in vivo and in vitro studies have demonstrated that BMMNCs can inhibit the production of IL-6, IL-1β, and TNF-α by the microglia and the secretion of anti-inflammatory cytokines such as IL-4, IL-10, and TGF-β1 [36–38]. Central effects involve the release of trophic factors such as cytokines, chemokines, and extracellular vesicles to improve outcomes after IS. Moreover, stem cells can induce angiogenesis and the repair of the blood–brain-barrier (BBB) following IS [39–42]. Furthermore, BMMNC therapy can play a crucial role in repairing and functionally reconstructing damaged neural circuits [43–46]. The safety of BMMNCs in stroke patients has been confirmed by several clinical trials [20, 47]. However, the efficacy of BMMNCs remains controversial.

In the present study, analysis of the three-month NIHSS score revealed a favorable result in patients who underwent BMMNC transplantation, indicating a better improvement of the neurological deficit over the short term. Of the five included RCTs, one study reported a significant difference between the BMMNC transplantation group and the control group; the others all showed improved outcomes in the BMMNC transplantation group, although the observed differences were not statistically significant. However, there was no statistically significant difference detected between the two groups at six months after treatment. An RCT conducted on acute IS patients revealed that at six months, the outcome of the BMMNC transplantation group was inferior to that of the control group [20]. The trophic factors and cytokines produced by BMMNCs may accelerate the recovery of IS [20]. Consequently, differences were evident in the first three months, although this impact was less prominent at six months; the mechanisms responsible for these effects remain unclear. With regard to the six-month mRS score and BI score, our findings suggest that BMMNC transplantation has the potential to enhance patient outcomes, although this was not statistically significant. This finding implies that BMMNC transplantation may not significantly improve overall disability, dependence, and activities of daily living when compared to the control group. However, it is important to note that substantial heterogeneity was observed in the BI score between the two included studies. Furthermore, we did not detect a significant difference in infarct volume when compared between the BMMNC transplantation groups and control groups. Prasad et al. [28] observed a reduction of infarct volume in the BMMNC transplantation group, while Moniche et al. [20] did not observe a significant change. Notably, the different baseline infarct volumes between the two trials may lead to different conclusions. Moniche [20] proposed that instead of brain infarct volume, other mechanisms could be used to predict functional outcomes in a more precise manner, such as the restoration of cortical connections between brain hemispheres. Therefore, the recommendation is to utilize advanced magnetic resonance imaging (MRI) techniques, such as diffusion tensor imaging or functional MRI, to validate the hypothesis of enhanced neuroplasticity.

Our meta-analysis revealed a significant change in the BI score with low doses of BMMNC treatment. A recently published multicenter RCT demonstrated that the efficacy of treatment was more prominent in the low-dose group (2 × 10^6^ BMMNCs/kg) when compared to the high-dose group (5 × 10^6^ BMMNCs/kg), with higher BI scores and lower NIHSS scores [20]. However, animal studies have shown that the administration of high doses of cells can increase the efficacy of cell-based therapy in stroke [48–50]. Yang et al. [50] observed a more effective treatment result when using BMMNCs at doses of 1 × 10^7^ and 3 × 10^7^ cells/kg when compared with 1 × 10^6^ BMMNCs/kg. This discrepancy could be attributed to the high dose employed in clinical trials, which is far less than that used in animal studies. Wang et al. [49] also found no significant difference between groups of animal models of stroke that were implanted with BMMNCs doses ranging from 1 × 10^6^ to 1 × 10^7^ BMMNCs/kg. However, exceeding the optimal threshold for transplanted cells may result in saturation of the damaged striatum, thus leading to a progressive reduction in the survival of transplanted cells due to insufficient nutrients.

With regard to the route of administration, our meta-analysis revealed that the subarachnoid pathway exhibited a greater propensity for improving NIHSS outcome, while the intra-arterial pathway demonstrated greater efficacy in terms of changes in BI score. However, the limited number of RCTs resulted in the inclusion of only a small number of patients in each subgroup, potentially impacting the validity of these findings. Some studies also compared the effect of BMMNC transplantation using intra-arterial and intravenous routes; however, these studies found no significant difference in terms of efficacy outcomes between the two methods [51, 52]. The intravenous route is regarded as the simplest and least invasive technique [53], although a considerable portion of transplanted cells can become trapped in peripheral organs [54, 55]. On the other hand, the intra-arterial route allows for more efficient biological biodistribution by bypassing peripheral filtering organs such as the liver, spleen, and lungs [56]. The subarachnoid route achieved the highest engraftment rate among all administration routes with approximately one-third of the cells still migrating to the ischemic area [57, 58]. As mentioned earlier, during the early stages of IS, BMMNC therapy contributes to functional recovery mainly by releasing trophic factors. Therefore, it is advisable to consider less invasive treatments, such as intra-arterial and intravenous transplantation [59]. However, during the chronic phase of IS, when the acute pathophysiological changes have stabilized, the focus shifts to the replacement of damaged tissue. In this case, the subarachnoid pathway or other invasive routes are more commonly used to directly deliver cells into the central nervous system, thus facilitating the reconstruction of the neural circuit, and the replacement of damaged brain tissues in IS [60, 61]. However, the use of subarachnoid delivery requires careful consideration since subarachnoid routes are associated with several problems, including invasiveness and the risk of intracranial infection [62]. Furthermore, the intranasal and intraperitoneal routes are currently in the preclinical study phase and require more evaluation of their safety and efficacy before being implemented in large-scale clinical trials.

This meta-analysis also has some limitations that need to be considered. Firstly, it should be noted that the sample size of the included RCTs was small; this may have led to reliability deficiencies. In addition, due to the lack of evidence, we did not investigate the optimal timing of BMMNC transplantation in this article. Notably, some RCTs did not use sham injections; furthermore, these trials did not keep participants and observers blinded due to the obvious differences between BMMNC transplantation injection and conventional medical therapy.

## Conclusion

Our meta-analysis showed that BMMNCs transplantation was safe, but its validity remains to be certified. In the future, more RCTs with long-term follow-ups are needed to validate the specific efficacy of BMMNC therapy in IS.

### Supplementary Information

Below is the link to the electronic supplementary material.

Supplementary file1 (PDF 20 KB)

## Data Availability

All data generated or analyzed during this study are included in this published article and its supplementary information files.
