# Supplementary material for: Safety and efficacy of bone marrow mononuclear cell therapy for ischemic stroke recovery: a systematic review and meta-analysis of randomized controlled trials
Source: Neurol Sci. 2024 Jan 3;45(5):1885–96. doi: 10.1007/s10072-023-07274-x (PMC11021295; doi:10.1007/s10072-023-07274-x)
Supplement: Supplementary file 1 — Supplementary file1 (PDF 20 KB) [file 10072_2023_7274_MOESM1_ESM.pdf]

Neurological Sciences

Safety and efficacy of bone marrow mononuclear cell therapy for ischemic stroke recovery: a systematic review and meta-analysis of randomized controlled trials

Yanbing Tang[#], Zilan Wang[#], Haiying Teng, Hanyu Ni, Huiru Chen, Jiaye Lu, Zhouqing Chen[*], Zhong Wang[*]

[#]Yanbing Tang and Zilan Wang contribute equally to this work.

[*]Corresponding author: Zhouqing Chen or Zhong Wang, the First Affiliated Hospital of Soochow University, 188 Shizi Street, Suzhou, Jiangsu Province, 215006, China. E-mail address: zqchen6@163.com or wangzhong761@163.com.

Table S1: Search strategies and results

Database1 MEDLINE

("stem cell"[Title/Abstract] OR "stem cells"[Title/Abstract] OR "bone marrow mononuclear cell"[Title/Abstract]) AND "stroke"[Title/Abstract] AND "random*"[Title/Abstract]

Database2 EMBASE

#1 'stem cell':ti,ab,kw OR 'stem cells':ti,ab,kw OR 'bone marrow mononuclear cell ':ti,ab,kw

#2 stroke:ti,ab,kw

#3 random*:ti,ab,kw

#4 #1 AND #2 AND #3

Database3 Cochrane

#1 ((TS=(stem cell)) OR TS=(stem cells)) OR TS=(bone marrow mononuclear cell)

#2 TS=(stroke)

#3 TS=(random*)

#4 #3 AND #2 AND #1

Clinicaltrial.gov

- Condition or disease: stroke
- Other terms: stem cell OR stem cells OR bone marrow mononuclear cell OR bone marrow mononuclear cells
